# Cardiac cAMP: production, hydrolysis, modulation and detection

**DOI:** 10.3389/fphar.2015.00203

**Published:** 2015-10-01

**Authors:** Cédric Boularan, Céline Gales

**Affiliations:** Institut des Maladies Métaboliques et Cardiovasculaires, Institut National de la Santé et de la Recherche Médicale, U1048, Université Toulouse III Paul SabatierToulouse, France

**Keywords:** GPCR, resonance energy transfer, phosphodiesterase, protein kinase A (PKA), Cyclic AMP

## Abstract

Cyclic adenosine 3′,5′-monophosphate (cAMP) modulates a broad range of biological processes including the regulation of cardiac myocyte contractile function where it constitutes the main second messenger for β-adrenergic receptors' signaling to fulfill positive chronotropic, inotropic and lusitropic effects. A growing number of studies pinpoint the role of spatial organization of the cAMP signaling as an essential mechanism to regulate cAMP outcomes in cardiac physiology. Here, we will briefly discuss the complexity of cAMP synthesis and degradation in the cardiac context, describe the way to detect it and review the main pharmacological arsenal to modulate its availability.

## Introduction

In cardiomyocytes, the influx of Ca^2+^ ions through voltage-dependent L-type Ca^2+^ channels (LTCC) plays an essential role in cardiac excitability and in coupling excitation to contraction of these cells. The depolarizing current through LTCC (ICa) contributes to the plateau phase of the cardiac action potential as well as to pacemaker activity in nodal cells (Shaw and Colecraft, 2013). This influx of Ca^2+^ triggers the release of intracellular stores of Ca^2+^ from the sarcoplasmic reticulum via the Ryanodine receptor (RyR), which results in activation of myofilaments contraction. Alterations in density or function of LTCC have been implicated in a variety of cardiovascular diseases, including atrial fibrillation (Van Wagoner et al., 1999) or heart failure (Mukherjee and Spinale, 1998). Cyclic adenosine 3′,5′-monophosphate (cAMP) is the main second messenger of the β-adrenergic receptor signaling inducing phosphorylation of the LTCC and the ryanodine receptor to increase the amount of intracellular Ca^2+^ necessary for heart contractility (responsible for positive chronotropic and inotropic effects during sympathetic stimulation) (Guellich et al., 2014). Moreover, catecholamine stimulated β-adrenergic receptor not only leads to cAMP effector dependent-troponin I phosphorylation to allow faster force development and shortening during systole and faster force relaxation and re-lengthening during diastole but also mediated cAMP effector dependent-phospholamban phosphorylation responsible for Ca^2+^ re-uptake in the sarcoplasmic reticulum and myofilament relaxation (lusitropic effects) (Bers, 2008). However sustained stimulation of this pathway may be detrimental thus leading to cardiac remodeling and development of heart failure (Brodde, 1993; Kiuchi et al., 1993). Thus, the proper physiological cardiac function relies on tight control of cellular cAMP concentration by fine-tuning the balance between cAMP synthesis and degradation. In mammalian cells, cAMP is produced by adenylyl cyclases (AC). Extracellular stimuli such as neurotransmitters, hormones, chemokines, lipid mediators, and drugs, can modulate AC activity to increase or decrease cAMP production by binding to a large number of transmembrane G protein-coupled receptors (GPCRs). The degradation of cAMP to AMP is catalyzed by phosphodiesterases (PDE) that are regulated by intracellular nucleotide concentrations, phosphorylation, binding of Ca^2+^/calmodulin and other regulatory proteins while cAMP efflux out of the cell is mediated by cyclic nucleotide efflux transporters. Over the years, several genetic models have been created to assess the role of the cAMP synthesis and hydrolysis proteins in cardiac physiology (Table 1). Once cAMP is produced it activates a set of diverse proteins, including cAMPdependent-protein kinase (PKA) or cAMP-dependent exchange proteins (Epac), the two main cAMP effectors to mediate downstream signaling as well as cyclic nucleotide-gated ion channels (CNGC) and POPDC proteins (Beavo and Brunton, 2002). From the compartmentation hypothesis proposed by Brunton et al., in which cAMP microdomains are distinctly coupled to cellular functions (Brunton et al., 1981), a variety of technologies has been developed to study *in vivo* the different localizations and organization around macromolecular complexes to ensure a fine-tuned spatio-temporal compartmentation of cAMP production (for detailed reviews (Baillie, 2009; Edwards et al., 2012; Perera and Nikolaev, 2013). Recently, these tools led to the identification of a β2-adrenergic-dependent cAMP compartmentation defect in failing cardiomyocytes (Nikolaev et al., 2010). In this review, we will focus on cAMP in synthesis and hydrolysis in cardiology, the way to detect it and how to manipulate this cAMP pathway.

**Table 1:** **Cardiac phenotype for cAMP synthesis, hydrolysis and transporter proteins adapted from Guellich et al. (2014)**.

**Class**	**Protein family**	**Substrate affinity**	**Protein**	**Cardiac function**	**Available model**	**References**
**AC**			AC1	Modulates If pacemaker current		Mattick et al., 2007
			AC5	Regulates contractility β-adrenergic dependent	AC5 Knockout	Iwamoto et al., 2003; Okumura et al., 2003a,b; Tang et al., 2006
				Myocardardial contractility increases LV function increases heart rate, reduces inotropic, lusitropic and chronotropic response to β1 AR	AC5 Transgenic	Tepe et al., 1999; Esposito et al., 2008; Lai et al., 2013
			AC6	LV systolic and diastolic dysfunction LV contractility increases with βAR stimulation enhanced contractile function	AC6 Knockout AC6 Transgenic	Gao et al., 1999, 2008; Phan et al., 2007; Tang et al., 2008, 2010; Guellich et al., 2010
			AC8	Enhances basal intrinsic contractility	Cardiomyocyte specific AC8 Transgenic	Lipskaia et al., 2000
			sAC	Apoptosis of coronary endothelial cells heart rate increase	ADCY10 knockout	Kumar et al., 2009; M.G. Informatics^1^
**PDE**	PDE1	1–100 μM	PDE1A	Cardiomyocyte hypertrophy Cardiac fibroblast activation and cardiac fibrosis		Miller et al., 2011
			PDE1B		PDE1B Knockout	Yu et al., 1997; M.G. Informatics^2^
			PDE1C		PDE1C knockout mice	Vandeput et al., 2007; M.G. Informatics^3^
	PDE2	30 μM	PDE2A	PDE2 expression is increased in experimental heart failure		
				L-type Ca2+ channel activity Contractility	PDE2A: embryonic death (El7)	Hartzell and Fischmeister, 1986; Fischmeister et al., 2005
	PDE3	0.08 μM	PDE3A	Regulates β-adrenergic signaling, cardiac contractility, pacemaking, and output reduces cardiomyocyte apoptosis and prevents ischemia/reperfusion induced myocardial infarction cardiac contractility, LTCC activity	PDE3A knockout mice Cardiomyocyte PDE3A overexpressing mice	Tarpey et al., 2003; Ding et al., 2005a,b; Sun et al., 2007; Molenaar et al., 2013; Iwaya et al., 2014
	PDE4	1–4 μM	PDE4A	?	PDE4A knockout mice	Jin et al., 2005
			PDE4B	Arrhythmogenesis	PDE4B knockout mice	Leroy et al., 2011
			PDE4D	β-adrenergic signaling RyR2 hyperphosphorylation, arrhythmia	PDE4D knockout mice	Lehnart et al., 2005; Bruss et al., 2008
	PDE7	0.03–0.2 μM	PDE7A	?	PD17A Knockout	Yang et al., 2003
	PDE8	0.04–0.8 μM	PDE8A	Regulation of LTCC Ca^2+^ signaling, Ryr2 Ca^2+^ load	PDE8A knockout mice	Patrucco et al., 2010
**Cyclic nucleotide efflux transporter**			ABCC4	Enhances contractility and cardiac hypertrophy	MRP4 Knockout mice	Sassi et al., 2012
			ABCC5	?		

*This table summarized the main cardiac functions and available model for the different class of proteins regulating cAMP availability: references for ACs (AC1, Mattick et al., 2007; AC5 KO, Iwamoto et al., 2003; Okumura et al., 2003a,b; Tang et al., 2006; AC5 Tg, Tepe et al., 1999; Esposito et al., 2008; Lai et al., 2013; AC6 KO, Tang et al., 2008, 2010; AC6 Tg, Gao et al., 1999, 2008; Phan et al., 2007; Guellich et al., 2010; AC8, Lipskaia et al., 2000; sAC, Kumar et al., 2009; M.G. Informatics), references for PDEs (PDE1A, Miller et al., 2011; PDE1B, Yu et al., 1997; M.G. Informatics; PDE1C, Vandeput et al., 2007; M.G. Informatics; PDE2A, Hartzell and Fischmeister, 1986; Fischmeister et al., 2005; PDE3A, (Tarpey et al., 2003; Ding et al., 2005a,b; Sun et al., 2007; Molenaar et al., 2013; Iwaya et al., 2014); PDE4A, Jin et al., 2005; PDE4B, Leroy et al., 2011; PDE4D, Lehnart et al., 2005; Bruss et al., 2008; PDE7, Yang et al., 2003; PDE8, Patrucco et al., 2010) and references for cyclic nucleotide transporter (ABCC4, Sassi et al., 2012)*.

## cAMP in the cardiac tissue

### cAMP synthesis

**Figure 1:**
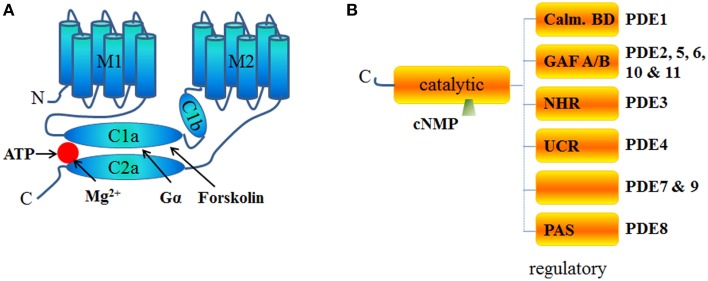
**Schematic structure of ACs and PDEs. (A)** Structure of Adenylyl cyclase is comprised of 2 transmembrane domains (M1 and M2 6 helixes each) and 2 cytosolic domains (C1 and C2) subdivided into a and b domains. C1 and C2 contain the catalytic core, the Gα and the forskolin binding sites and other regulatory sites. C2b domain is almost inexistent in all AC isoforms. **(B)** PDEs are homodimers with the exception of PDE1 and PDE6 (usually heterotetramers). PDEs have an NH_2_-terminal regulatory domain and share a conserved catalytic domain located in the COOH-terminal portion of the protein. The structure of the regulatory domain varies according the PDE isoform. GAF is an acronym for cGMP-specific PDE, Adenylyl cyclases and FhlA, NHR for N-terminal Hydrophobic Region, PAS for Per-ARNT-Sim and Calm. BD for calmodulin binding domain. No known domains are present in PDE7 or PDE9 regulatory C-terminal part. PDE4 proteins are classified as “long” or “short” isoforms, depending on the presence or absence of two highly conserved domains, Upstream Conserved Region 1 (UCR 1) and Upstream Conserved Region 2 (UCR 2) which interact to form a regulatory module that may influence catalytic activity by a PKA-dependent phosphorylation mechanism (Houslay, 2001; MacKenzie et al., 2002).

**Figure 2:**
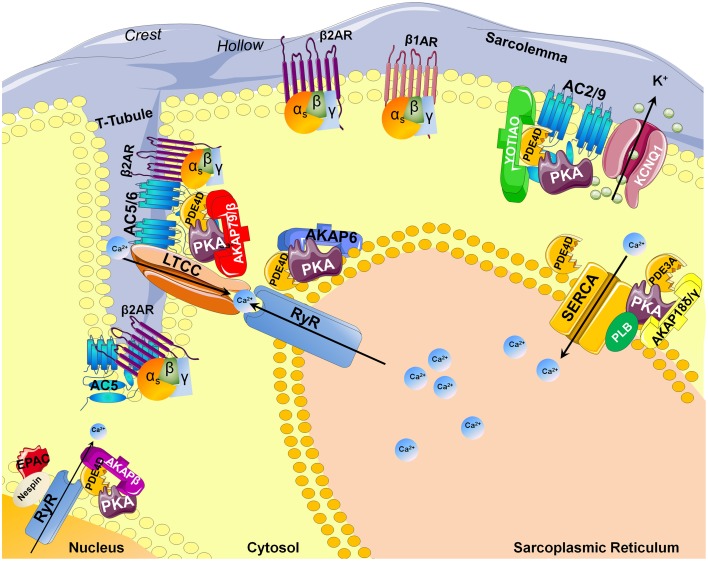
**AKAP-dependent AC and PDE compartmentalizations in the cardiomyocyte**. Abbreviations stand for: AKAP, A-kinase anchor proteins; PKA, Protein Kinase A; β2AR/β1AR, beta adrenergic receptor; PLN, Phospholamban; EPAC, cAMP-dependent exchange proteins; AC, Adenylyl cyclase; RyR, Ryanodine Receptor; SERCA, sarco/endoplasmic reticulum Ca^2+^-ATPase; KCNQ1, potassium channel voltage gated KQT-like subfamily Q; PDE, Phosphodiesterase; T-tubule, Transverse tubule; LTCC, L-type calcium channel.

Adenylyl cyclases (AC) are ubiquitous enzymes that catalyze the conversion of Adenosine triphosphate (ATP) into cAMP and pyrophosphate. ACs structure consists in 12 transmembrane domains divided into 2 hydrophobics domains (6 transmembrane domains each) and 2 main intracellular loops called C1 and C2 that naturally dimerize to form the catalytic domain (Figure 1A). In mammals, 9 transmembrane and 1 soluble AC (sAC) encoded by different genes have been identified and have different regulatory mechanisms (Willoughby and Cooper, 2007). Mammalian ACs are strongly activated by Mn^2+^ or Mg^2+^ (Tesmer et al., 1999) and inhibited by millimolar concentrations of free Ca^2+^ probably acting as a Mg^2+^ competitor (Mou et al., 2009) but at submicromolar concentrations, Ca^2+^ can activate AC via calmodulin (CaM) throught its binding to a putative helical structure on the C1b region (Halls and Cooper, 2011). More precisely AC1, 3, and 8 are Ca^2+^/CaM sensitive isoforms which localize in lipid rafts while AC2, 4, 7, and 9 are Ca^2+^/CaM insensitive and are excluded from these membrane domains (Willoughby and Cooper, 2007). On the contrary, biochemical studies on membrane preparations revealed that AC5 and AC6 could be inhibited by Ca^2+^ (independently of CaM) in the submicromolar range (Guillou et al., 1999; Hu et al., 2002). Along with nitric oxide (NO), Hydrogen sulfide (H2S) is a biological gaseous transmitter able to modulate cAMP production. Using NO donors, the gasotransmitter NO is thought to attenuate forskolin-stimulated AC5 and AC6 isoforms activities without altering their basal activity on membrane from rat striatum (Hudson et al., 2001). Even if H2S is a poisonous gas used as a chemical reagent, H2S is endogenously formed in mammalian cells from cysteine by the action of cystathionine β-synthase with serine as a by-product at a concentration around 50–160 μmol/L (Goodwin et al., 1989). In the central nervous system, H2S enhances NMDA receptor-mediated response via cAMP production (Kimura, 2000) while in cardiac context it can also suppressed AC activity and, therefore, decreased forskolin-stimulated cAMP accumulation in different cell lines and tissue (Lim et al., 2008; Yong et al., 2008). In the cardiomyocytes, expression of AC1, AC5, AC6, AC8, and sAC has been detected and most function as modulators of inotropic and chronotropic β-adrenergic receptor (β-AR) signaling output (Table 1) but AC5 and AC6 represent the dominant isoform (Defer et al., 2000). Along with AC distribution within membrane microdomains (Efendiev and Dessauer, 2011), cAMP synthesis is spatially restricted by localization of activating receptors like β1-adrenergic receptors or β2-adrenergic receptors at caveolae or non-caveolae plasma membrane domains (Rybin et al., 2000; Ostrom et al., 2001). Moreover, specific A-kinase anchor proteins (AKAP) complexes (Kapiloff et al., 2014) have been identified as a potential molecular mechanism for the formation of specific cAMP microdomains (Kapiloff et al., 2014). The AKAPs constitute signaling hub proteins that scaffold on a same membrane domain the AC and the regulatory subunit of protein kinase A (PKA) cAMP effector, thus confining the enzyme activity to discrete locations within the cell. Cardiac myocytes exhibit at least four distinct AKAP complexes: AKAP79/150 (aka AKAP5) with AC5/6 (Nichols et al., 2010); mAKAPβ (aka AKAP6) with AC2/5 (Kapiloff et al., 2009), YOTIAO (aka AKAP9) with AC2/9 (Piggott et al., 2008), and AKAP18δ with PKA (Fraser et al., 1998) (Figure 2).

### cAMP elimination: Phosphodiesterases and cyclic nucleotide efflux transporters

#### Phosphodiesterases

Cyclic AMP is hydrolyzed exclusively by cyclic nucleotide PDEs classified in 11 families and encoded by at least 21 different genes with the existence of some splice variants (Omori and Kotera, 2007). PDEs are structured around a catalytic domain containing the cyclic nucleotide binding site conserved across all families and a regulatory N-terminus varying according to the different PDEs (Figure 1B). In the heart, 8 PDE families have been described: PDE1; PDE2, PDE3, PDE4, PDE5, PDE7, PDE8, and PDE9. Among them, PDE1, PDE2, and PDE3 are dual-specificity enzymes that can hydrolyze both cAMP and cGMP while PDE4, PDE7, PDE8 selectively hydrolyze cAMP and conversely PDE5, PDE9 selectively hydrolyze cGMP. Of the cAMP-hydrolyzing PDEs expressed in the heart, cGMP inhibits PDE3 and possibly PDE1, whereas PDE2 is activated by cGMP (detailed review in Zaccolo and Movsesian, 2007). Jurevicius and Fischmeister (1996a,b) provided the first direct evidence for PDE-mediated cAMP signaling compartmentation, showing that PDE inhibition allowed local β-adrenergic stimulation to enhance Ca^2+^ currents in frog ventricular myocytes (Jurevicius and Fischmeister, 1996a,b). Later on, imaging approaches confirmed that PDEs play a key role in shaping the intracellular cAMP gradient in rat neonatal cardiomyocytes (Zaccolo et al., 2000; Zaccolo and Pozzan, 2002). Like ACs, PDEs have also been shown to be compartmented by AKAPs complexes. Thus, specific cAMP hydrolysis-based PDE4 enzyme were shown to interact with mAKAP for PDE4D3 (Dodge et al., 2001); AKAP9 for PDE4D3 (Taskén et al., 2001), AKAP95 (aka AKAP8) for PDE4A (Asirvatham et al., 2004), AKAP149 for PDE4A (Asirvatham et al., 2004) (Figure 2).

#### Cyclic nucleotide efflux transporters

In addition to PDEs and ACs, the intracellular concentration of cAMP is regulated by its efflux into the extracellular space through a specific transmembrane transport system named multidrug resistance proteins (MRP) (Cheepala et al., 2013) that belongs to the ATP-binding cassette (ABC) transporter superfamily (subfamily C). Three of them (MRP4 aka ABCC4, MRP5 aka ABCC5, and MRP8 aka ABCC11) have the ability to actively extrude cAMP and cGMP from the cell (Kruh and Belinsky, 2003) and in cardiac myocytes, MRP4 has been shown to enhance cAMP formation, contractility, and cardiac hypertrophy (Sassi et al., 2012). The compartimentation of MRPs expression may also play an important role in the intra- and extracellular cAMP signaling processes. For instance, caveolin-rich membrane MRP4 localization (Sassi et al., 2008) could explain the local MRP4-modulated contraction of cardiac myocytes induced by activation of β-adrenoceptor (Sellers et al., 2012).

### cAMP in heart failure

Heart failure (HF) occurs when the heart is unable to pump sufficiently to maintain blood flow to meet the body's needs. Around 2% of adults have HF and this percentage increases to 6–10% for people over the age of 65 (McMurray and Pfeffer, 2005). The HF syndrome arises as a consequence of an abnormality in cardiac structure, function, rhythm, or conduction. As stated in introduction, cAMP primarily, but not exclusively, controls beating frequency, force of contraction and relaxation, essentially through the β-adrenergic signaling pathway. This pathway is necessary for the beneficial effects of catecholamines on cardiac contractility. During heart failure set up, increased sympathetic activity drives the βAR overstimulation in cardiomyocytes, thus promoting higher intracellular cAMP signals for compensatory cardiac function in the heart (Baker, 2014). However, chronic βAR stimulation and uncontrolled cellular cAMP signals have been shown to affect heart function in a much more detrimental way responses such as cell apoptosis (Zhu et al., 2003) and the loss of pump function (Michel et al., 1990; Engelhardt et al., 1999; Lohse et al., 2003), ultimately leading to HF setup. During the ongoing of the disease, a down-regulation of β1AR expression (Nikolaev et al., 2010) is correlated with a modulation of Gαi proteins (Eschenhagen et al., 1992a,b) expression to attenuate cAMP synthesis. The ratio between β1AR and β2AR converts the latter to be the major βAR subtype in failing hearts. Interestingly, associated with this receptor expression imbalance, the β2AR dominant-induced cAMP signal is broadly distributed in the failing heart (Nikolaev et al., 2010) compare to a compartmentalized cAMP signal in physiological condition. The functional output of this broadly distributed cAMP signal in modulating contractile properties in failing hearts has to be studied. In failing cardiomyocytes, chronic β2AR stimulation also promotes CaMKII-dependent contractile responses which has a pronounced role in promoting the development of cardiac hypertrophy, myocyte apoptosis, cardiac dysfunction and arrhythmias by causing sarcoplasmic reticulum Ca^2+^ overload (Anderson et al., 2011). Inhibition of CaMKII is able to ameliorate cardiac remodeling and reduce cardiac arrhythmias after myocardial infarction (Zhang et al., 2005). Although the direct link between cAMP and CaMKII is still missing, the detrimental CaMKII activity in cardiomyocytes could be related to dysregulation of distribution of cAMP signals under chronic βAR stimulation. HF is a complex process where the various components in the cAMP signaling pathway constitute potential pharmacological targets.

## Modulation of cAMP concentration in the cardiac tissue

### Modulation of cAMP production

#### Targeting adenylyl cyclases

##### Pharmacological AC activators

The most prominent AC activator is forskolin (FSK). FSK, a diterpene extracted from the plant *Coleus forskohlii*, directly activates all AC isoforms except AC9. Despite a strong hydrophobic property, its action is not limited to the native membrane-bound form of the enzyme since it can readily stimulate some synthetic soluble ACs. FSK binds to the same cleft that contains the active site of AC (Tesmer et al., 1997) where it glues together its two cytoplasmic domains (Figure 1A) by a combination of hydrophobic and hydrogen-binding interactions (Zhang et al., 1997). Based on equilibrium dialysis experiments of the C1 and C2 domains of type AC5 and AC2, respectively, the C1/C2 complex binds only one Gsα, one ATP, and one FSK molecule (Dessauer et al., 1997). However, FSK has been shown to also inhibit a number of membrane transport proteins and channel proteins like Glucose transporter or voltage dependent K^+^ channel (Laurenza et al., 1989). As Protein kinase C (PKC) activates AC2 by phosphorylating it on Thr-1057 (Böl et al., 1997), another alternative, but more restrictive one to activate AC, relies on the use of Phorbol 12-myristate 13-acetate (PMA) a phorbol diester and a potent tumor promoter known to activate PKC signaling. Thus, PKC-dependent phosphorylation of AC-C1 domain induces AC activation (Ebina et al., 1997). However, one must be cautious on the use of PMA as a specific AC-activator since PMA has also been reported to have actions on non-kinase proteins including chimaerins, RasGRP, and Unc-13/Munc-13 (Han and Meier, 2009; Kazanietz et al., 1995).

##### Pharmacological AC inhibitors

As schemed in Figure 1A, ACs are structured around 2 hydrophobic domains and 2 main intracellular loops containing the catalytical domain and the diterpene regulatory site. Based on this structure, ACs inhibitors can be divided into 4 groups (reviewed in Seifert et al., 2012): (i) the inhibitors competing with the ATP at the catalytic site like MANT-GTP (Gille and Seifert, 2003), (ii) the uncompetitive P-site inhibitors like 2′,5′-dideoxyadenosine-3′-tetraphosphate Vidarabine [aka 9-β-D-arabinofuranosyladenine (ara-A)] (Seifert, 2014) or NKY80 (a cell-permeable quinazolinone) to name a few, which work by stabilizing a pyrophosphate-bound transition state (Dessauer et al., 1999; Onda et al., 2001), (iii) the allosteric non-competitive inhibitors targeting the diterpene regulatory site like BODIPY-FS in presence of divalent cations (Erdorf et al., 2011), and (iv) the allosteric non-competitive inhibitors targeting alternated and unknown site like calmidazolium (Haunsø et al., 2003). Even though some specificity has been assigned to some of the molecules listed, to our knowledge, those inhibitors have not been accurately examined at all ACs isoforms, thus preventing any formal conclusion only based on their use to assess the involvement of AC activity.

#### Targeting G-protein coupled receptor signaling

We previously mentioned that cAMP constitutes the master second messenger of β-adrenergic receptor signaling which belong to the G protein-coupled receptors (GPCRs) family. According to conventional knowledge, 7 transmembrane GPCRs at the plasma membrane convert extracellular signals into intracellular ones through canonical heterotrimeric G proteins which transduce signals from GPCRs to secondary effectors thus leading to the second messengers production and the propagation of the signal through ensuing regulation of numerous downstream intracellular signaling targets (Gilman, 1987). G proteins localized on the cytoplasmic side of the plasma membrane and are composed of a guanine nucleotide binding α subunit (Gα) and a βγ dimer (Gβγ), both constitutively associated in the G protein inactive state. Upon GPCR activation, the Gα*βγ* protein associates with the receptor thus allowing GDP/GTP exchange on the Gα GTPase domain, leading to subsequent Gα-GTP and Gβγ dissociation both regulating downstream specific signaling targets (Denis et al., 2012). Intrinsic GTPase activity of the Gα then allows GTP hydrolysis and to turn off the G protein activity to its initial inactive Gα*βγ* associated state. G proteins have been classified into five subfamilies (Gi/o, Gs, Gq/11, and G12/13) according to the secondary effector of the Gα subunit (Denis et al., 2012). Thus, isoforms of the Gαi/o family classically inhibit ACs and cAMP production while, conversely, isoforms from the Gαs family activate ACs to favor cAMP production. It follows that modulation of the activity of cardiac expressed Gαi- or Gαs-coupled receptors either through the use of selective GPCR agonists and antagonists or G proteins activators or inhibitors will directly alter the G protein activity and cAMP availability.

##### GPCR agonists and antagonists

In the human genome, it is estimated that the GPCR superfamily consists in ~600–1000 receptors (Lander et al., 2001; Vassilatis et al., 2003; Fredriksson and Schioth, 2005) where ≈ 200 have known cognate agonists and the larger part are still “orphan,” i.e., without yet identified agonists (Vassilatis et al., 2003). Evaluation of GPCR expression *in vivo* has been largely hampered by lack of specific antibodies against this class of receptors. Thus, over the years, microarray technology allowed researchers to monitor the mRNA expression levels of thousands of GPCRs encoding genes. Based on the available genomic data (Hakak et al., 2003; Katugampola and Davenport, 2003; Tang and Insel, 2004; Regard et al., 2008; Moore-Morris et al., 2009), we tried to summarize the different GPCRs detected in the whole cardiac tissue (cardiomyocytes, endothelial cells, fibroblasts…) (Table 2), their classical G protein coupling and a selective agonist/antagonist for most of them. This list is non-exhaustive and selectivity or description of these compounds will not be detailed here. Thus, selective pharmacological targeting of Gαi- or Gαs-coupled cardiac receptors represents a way to modulate intracellular cAMP levels. It is noteworthy that the classical GPCR coupling has to be enlarged as a recent study shows dual agonist occupancy of the AT1-R and α2C-AR heterodimer, two GPCRs known to be coupled to Gαq and Gαi, respectively, created an original conformation different from the active individual protomers and triggered an atypical Gs/cAMP/PKA signaling (Bellot et al., 2015). Thus, co-stimulation or bivalent ligand development might be a new pharmacological area to regulate cAMP signaling (Berque-Bestel et al., 2008; Lezoualc'h et al., 2009).

**Table 2:** **GPCR expressed in heart: Gα coupling and pharmacological way to modulate their signaling**.

**Receptor**	**Subtype**	**Coupling**	**Example of agonist**	**Antagonist**
α-Adrenergic	la, lb, 1D,	Gq	Phenylephrine, Methoxamine	Corynanthiane, Prazosin
	2a, 2c	Gi	UK14304, B-HT92O	Yohimbine, RX821002
β-adrenergic	β1, β2, β3	Gs, Gi	Isoproterenol	Alprenolol, Pindolol, Propranolol
Adenosine	Adoral,	Gi	CHA, CPA	CPX, CPT, N-0840
	2a,2b	Gs	CGS21680, DPMA, HENECA	KW6002, Alloxazine, SCH-58261
Adrenomodulin	CGRP	Gi, Gs		
Angiotensin	AT1a	Gq, Gi	AngII, AngIII L162313	Losartan, Candesartan
	AT2	Gi	AngII, AngIII CGP42112A	PD123319, L-159686
Apelin	APJ	Gi	Apelin13	
	AVPR1a	Gq	vasopressin	Relcovaptan
Bradykinin	Bdkrbl, 2	Gq	Lys-BK	B9430
Calcium sensing	Ca-SR	Gs		
Cannabinoid	CB1	Gi	THC, CP-5594O, Nabilone	SR141716A, AM25l
Chemokine receptor	CX3CR1,	Gi	Fractalkine	
	CXCR2	Cri	IL-8, GCP2	
	CXCR4,7	Gi	SDF1α	AMD3100
	CXCR6	Gi	CXCL16	
	CCBP2,	Gi		
	CCR1, 5	Gi	MIP1α	
	CCR2	Gi	MCP-1	
	CCR10	Gi	CTACK	
	XCR1	Gi	xCL1	
Complement component receptor	C3aRl, C5R1	Gi		
Corticotropin releasing hormone	CRHR2	Gs	CRF, UCN1	Astressin
Cysteinyl leukotriene	Cystl1	Gq	LTD4	Cinalukast
Dopamine	Drd2	Gi	U-91356A, TNPA	L-741626
	Drd3	Gi	PD128907, BP897	Nafadotride, GR103691
Endothelin	ET-A, ET-B	Gq	ET1	PD142893
Frizzled	Fzdl, 2, 3, 5, 6, 7, 8			
Galanin	GalR2	Gq	Galanin, GALP	Galantide
GLP1		Gs		
Glucagon		Gq, Gs		
Gonadotropin receptor	LGR6, 7	Gi, Gq		
GPCR5	Raig2, GPCR5c	?		
Growth hormone secretagogue-receptor	GHS-R1a	GS, Gq		
Histamine	H1	Gq		Pyrilamine
	H2	Gs	Amthamine	Cimetidine
	H3	Gi	Immethridine	Ciproxifan
Latrophilin	Lphn1, 2	011-15	alpha-Latrotoxin	
Mas	Mas1, GPR168	?		
Melanin concentrating hormone	SLC-1	?	MCH	SNAP794l
Melanocortin	MC3R	Gs	γ2-MSH	SHU9119
Melatonin	MT1	Gi, Gq	Melatonin, S20098	Luzindole
	MT2	Gi	Melatonin, S20098	Luzindole
Muscarinic	M2, M3	Gi, Gq	Bethanecol, Xanomeline, Metoclopramine	Gallamine, Atropine, Scopolamine
Neuromedin U	NMUl, NMU2	Gi, Gq	NMU	
Neuropeptide Y receptor	NPY1, 2	Gi	NPY	BIBP3226, BIIE0246
Nucleotide	P2Y1	Gq	2MeSADP	BzATP, Suramin
	P2Y2	Gq, Gi	UTPγs	Suramin
	P2Y4	Gq, Gi	UTP	ATP
	P2Y5	Gi, G12/13		
	P2Y6	Gq	UDP	Suramin
	P2Y11	Gq, Gs	BzATP	Suramin
	P2Y13	Gi	2MeSADP	Ap4A
	P2Y14	Gi	UDP-glucose	
Opioid	MOP	Gi	DAMGO	Cyprodime
	DOP	Gi	DPDPE	Naltrindole
	KOP	Gi	Enadoline	GNTI
Opsin	Opn4	Gi, Gq		
Oxytocin	OXTR	Gq	Oxytocin, Carbetocin	
P518RF amide	SP9155	?	P518	
Vasoactive Intestinal Peptide receptor	VIPR2	Gs, Gq	VIP	acetyl-His-PheLysArg-VIP-GRF
PAR	PAR1	Gi, Gq, G12/13	Thrombin, Trypsin	BMS200261
	PAR2	Gi, Gq	Trypsin	
	PAR4	Gq	Thrombin, Trypsin	t-cinnamoylYPGKF
Platelet-activating factor receptor	PTAFR	Gq	PAF	Israpafant
Prolactin releasing peptide	GR3			
Prostacyclin	Ptgir	Gs		
Prostanoid	EP1	Gq	Iloprost	SH-19220
	EP4	Gs	ONO-AE1-734	AH23848
Relaxin-H2	LGR7, Rxfp4	Gi		
Serotonin	5HTR1a	Gi	R(+)-8-OH-DPAT	Spiperone
	5HTR1b	Gi	Sumatriptan, CGS12066	GR55562, SB216641
	5HTR2b	Gq	BW723C86	YM348
	5HT4	Gs	BIMU8	GR113808
Smoothened	Smoh	Gi		
Somatostain	SSTR3	Gi	L-796778	NVP-ACQ090
	SSTR4	Gi	NNC26-9100	s
Sphingosine	Edg1	Gi	S1P, FTY720-P	VPC23019
	Edg5	Gq,Gi G12/13	S1P	JTE-013
	Edg3	Gq, Gi, G12/13	S1P, FTY720-P	VPC23019
LPA	Edg2	Gi, G12 13	1-oleyl-LPA	VPC32183
Substance P	NK-1	Gq	substance P	GR-82334
Thromboxan	Tbxa2r	Gq	Thromboxan	Seratrodast
Urotensin	GPR14	Gq	UII	[Cha^6^]U-II_(4-11)_
**Other GPCR expressed in Heart**				
CD97	GPR77(C5L2)			
ELTD1	GPR82			
EMR1	GPR107			
TM7SF3	GPR108			
GPR1	GPR116			
GPR10	GPR120			
GPR17	GPR124			
GPR2l	GPR125			
GPR22	GPR133			
GPR27	GPR135			
GPR30	GPR137			
GPR31	GPR137b			
GPR34	GPR146			
GPR4	GPR153			
GPR44	GPR161			
GPR48	GPR175			
GPR54	GPR182			
GPR56	GPR183 (Ebi2)			

*This table summarized the list of mRNA encoding for GPCRs detected in the whole cardiac tissue (cardiomyocytes, endothelial cells, fibroblasts…) extracted from (Hakak et al., 2003; Katugampola and Davenport, 2003; Tang and Insel, 2004; Regard et al., 2008; Moore-Morris et al., 2009), the main known Gα protein coupling, an example of an agonist and an antagonist (this list is non-exhaustive and selectivity for each molecule is not discussed)*.

##### Gα activators

Cholera toxin (CTX) is a specific Gαs potent activator secreted by the bacteria *Vibrio cholerae* which catalyzed the ADP-ribosylation of the Gαs proteins. The ADP-ribosylation blocks the Gαs catalytic activity and thus prevents the Gαs subunit to hydrolyze the GTP once activated, leading to the ensuing sustained Gs and AC activity (De Haan and Hirst, 2004). CTX administration in non-ischemic or ischemic heart contributes to the genesis of arrhythmia highlighting the essential role for Gαs in the regulation of cardiac physiology (Huang and Wong, 1989). More recently, *Pasteurella multocida* toxin (PMT), produced by toxigenic strains of the Gram-negative *Pasteurella multocida* bacteria, was identified as a potent and selective activator of Gαq, Gαi, and Gα13 by deamidating a glutamine residue in the switch II region of the Gα-GTPase domain (Orth et al., 2005, 2008). It was recently shown that, *in vivo*, PMT treatment in mice increased secretion and expression of connective tissue growth factor (CTGF) in cardiac fibroblasts to aggravate cardiac hypertrophy and fibrosis (Weise et al., 2015).

##### Gα inhibitors

Basically, all Gα subunits inhibitors share a common molecular mechanism by preventing the GDP/GTP exchange on the Gα-GTPase domain. A famous specific and highly effective Gαi inhibitor is Pertussis Toxin (PTX). PTX is a protein complex released by the bacterium *Bordetella pertussis* in an inactive form. PTX catalyzes the ADP-ribosylation of the Gαi subunit of the heterotrimeric G protein. The Gαi subunit remains locked in its GDP-bound inactive state, thus unable to interact with the receptor and to inhibit adenylyl cyclase activity (Hsia et al., 1984; Burns, 1988). PTX-pretreatment is classically used to delineate the involvement of Gαi-dependent signaling. It revealed for instance an increase in β-AR dependent inotropic response and cAMP accumulation in isolated ventricular cardiomyocytes (Melsom et al., 2014), confirming the dual coupling of β2-AR to both Gαi and Gαs (Xiao, 2001) in the cardiac tissue. On a purified Gα activity assay, suramin, an antimicrobial drug, was identified as a more selective inhibitor for Gαs (IC_50_ ≈ 250 nM) than for Gαo (IC_50_ ≈ 2 μM) or Gαi (IC_50_ ≈ 5 μM). Suramin exerts its effects by binding the effectors binding site on the Gα proteins (Freissmuth et al., 1996). It has to be noted that suramin is a large highly sulfonated and negatively charged molecule that limits its use to *in vitro* studies as it cannot cross the cell plasma membrane. Hohenegger and coworkers worked on suramin derivatives to increase specificity toward Gαs and identified two compounds (NF449 and NF503) that suppress the Gαs activation coupled to β-adrenergic receptors, whereas they affect the Gαi/Gαo- and Gαq-coupled receptors (A1-adenosine and angiotensin II receptor, respectively) to a much lesser extent (Hohenegger et al., 1998). Lately, BIM-46174 and BIM-46187 were classified and used as pan Gα inhibitors targeting Gαs, Gαq/11, Gαi/o, and Gα12/13 family (Prévost et al., 2006). However, these cell permeable compounds have not be tested toward all the individual members of Gα subunit family and more recently Kostenis and colleagues found that BIM-46187 was more selective to inhibit Gαq depending on the cellular context (Schmitz et al., 2014).

##### Gβγ complex inhibitors

Smrcka and coworkers described small molecule Gβγ inhibitors that selectively block Gβγ-binding interactions to their effectors, including M119 and its highly related analog, gallein (Lehmann et al., 2008). These compounds blocked interaction of Gβγ and GRK2 *in vitro* and reduced β-AR–mediated membrane recruitment of GRK2 in isolated adult mouse cardiomyocytes (Casey et al., 2010). The authors showed M119 enhanced both adenylyl cyclase activity and cardiomyocyte contractility in response to β-AR agonist (Casey et al., 2010). More recently, in a screen for the identification of OXE receptor antagonists, Gue1654 was discovered as a biased inhibitor that selectively prevent Gβγ signaling without affecting the Gα pathway (Blättermann et al., 2012). The molecular mechanism underlying Gue1654 action is still under investigation.

#### Targeting tyrosine kinase receptor signaling

It has to be noted that AC have been involved in the mechanisms of action of insulin and other peptides of the insulin superfamily like Insulin-like Growth factor I, relaxin and mollusc insulin-like peptide which are ligands for tyrosine kinase receptors (TKR) (Pertseva et al., 2003). Earlier, it was shown that in the heart EGF, another TKR, triggered some AC mediated effect (Nair and Patel, 1993). At a molecular level, TKR dependent activation of AC can rely on the activation of PI3K, PKCζ, or the Gβγ complex (Wilson et al., 1996; Standaert et al., 1997; Molina-Munoz et al., 2006). Thus, modulating activities of TKRs and their signaling regulators constitute an alternative approach to modulate cAMP but such compounds will not be described in this review.

### Modulation of cAMP degradation

#### Phosphodiesterases inhibitors

The cardiostimulatory action of PDE make their inhibition as a promising therapeutic approach for the treatment of heart failure by sustaining cAMP production and action. Methylated xanthines, like theophylline, caffeine, or Iso-butyl-methyl-xanthyl (IBMX), are long known to act as competitive nonselective PDE inhibitors (Hess et al., 1975) but they also exhibit nonselective PDE action like adenosine receptor antagonist activities (Ukena et al., 1986). Over the years, several more specific and selective PDEs inhibitors have been developed. Representative selective inhibitors that can be used in cardiac tissue are listed below. Originally, 8-MM-IBMX was thought to be PDE1 selective (Rybalkin et al., 2002), but an extensive *in vitro* study characterized more potent and more selective compounds able to inhibit PDE1 activity like SCH51866 (Dunkern and Hatzelmann, 2007). The first specific inhibitor developed for PDE2 was EHNA [erythro-9-(2-hydroxy-3-nonyl)adenine] with an IC50 value of ~1 μM (Podzuweit et al., 1993) but a screen of compounds developed by Bayer showed that BAY60-7550 (an EHNA analog) was 100-fold more potent and 50-fold more selective for PDE2A over other PDEs compared to EHNA (Boess et al., 2004). Cilostamide-dependent PDE inhibition was discovered in 1970's (Hidaka et al., 1979) but cilostamide and its derivative selectivity for PDE3 family was described by Sudo et al. (2000). The prototypical PDE4 inhibitor is rolipram; originally named ZK62711, that was discovered in 1976 (Schwabe et al., 1976) but its use was limited by its associated side effects, particularly those affecting the gastrointestinal tract (Barnette and Underwood, 2000). Thus, in 2010, potency and selectivity of roflumilast and its active metabolite have been studied for all PDE (Hatzelmann et al., 2010). Roflumilast does not affect PDE enzymes apart from PDE4 family, and has a subnanomolar inhibitor activity toward all PDE4 splicing variants tested (Rabe, 2011). As the PDE4 family is encoded by 4 genes (PDE4A, B, C, or D) and 27 splice variants, identification of selective PDE4 subtypes inhibitors has been boosted and especially for PDE4B that can be selectively inhibited for example by triazine derivative (Hagen et al., 2014). ASB16165 was characterized as a specific and highly potent inhibitor for PDE7A with an IC50 value of 15 nM for human PDE7A (Kadoshima-Yamaoka et al., 2009). PDE8s are inhibited by dipyridamole, despite this drug is also known as a relatively nonselective cGMP specific PDE5 inhibitor (Soderling et al., 1998) while two studies have described a newly available PDE8 inhibitor developed by Pfizer, PF-04957325 (Vang et al., 2010; Shimizu-Albergine et al., 2012).

#### Cyclic nucleotide efflux transporters: description and inhibitors

As mentioned earlier, ABCC [ATP-binding cassette (ABC) transporter superfamily (subfamily C)] regulates cAMP efflux into the extracellular space to decrease cAMP availability. Three of them (ABCC4, ABCC5, and ABCC11) are expressed in cardiac tissue but ABCC4 is the most studied and has been shown to enhance cAMP formation, contractility, and cardiac hypertrophy (Sassi et al., 2012). Non selective inhibitors including MK-571, dipyrimamole or indomethacin (Reid et al., 2003) have been described to dually inhibit ABCC transporters and PDEs (Xie et al., 2011). Thus, the interpretation of experiments using those compounds has to take in account their side activities. In 2014, a high throughput screening identified Ceefourin 1 and 2 as highly selective ABCC4 inhibitors (Cheung et al., 2014). The authors described a micromolar inhibition of ABCC4 over other members of ABCC transporter families but no data are available concerning their effect on PDE activity (Cheung et al., 2014).

### Optogenetics methods to modulate cAMP availability

The genome of *Beggiatoa*, a sulfide-oxidizing bacterium, revealed the presence of a DNA sequence encoding for a cytosolic adenylyl cyclase directly linked to a BLUF (blue light receptor using FAD) type light sensor domain. This photoactivatatable adenylyl cyclase (bPAC) shows a low cyclase activity in the dark but that increases about 300-fold upon light activation (Stierl et al., 2011). Efetova et al. pioneered the use of bPAC to distinguish between the functions of alternative cAMP effectors in the *in vivo* regulation of a *Drosophila melanogaster* physiological process (Efetova et al., 2013) while Von Zastrow's group recently used the bPAC fused to different targeting sequences to assess the role of cAMP compartmentation in GPCR signaling (Tsvetanova and von Zastrow, 2014). Recently, a red light-activated PDE was engineered by recombining the photosensor module of *Deinococcus radiodurans* bacterial phytochrome with the effector module of *Homo sapiens* PDE2A (Gasser et al., 2014). Compare to the bPAC system, the red-shifted activation of this new tool will allow the creation of interesting animal model to study the spatio-temporal cAMP signaling pathway. This concept was declined for multiple targets referenced and collected by the CHROMus project (Shui et al., 2014) and applied to GPCR where the cytosolic part of the Rhodopsin receptor was replaced by the β2-AR receptor part to create a photoactivable Gαs coupled GPCR (Airan et al., 2009).

### Modulation of cAMP effectors

cAMP has four direct intracellular targets: protein kinase A (PKA), the exchange protein activated by cAMP (EPAC), the cyclic nucleotide gated ion channels (CNGC) and the popeye domain containing protein (POPDC). cAMP output signaling can be modulated by targeting its effectors.

#### PKA inhibitors and activators

**Figure 3:**
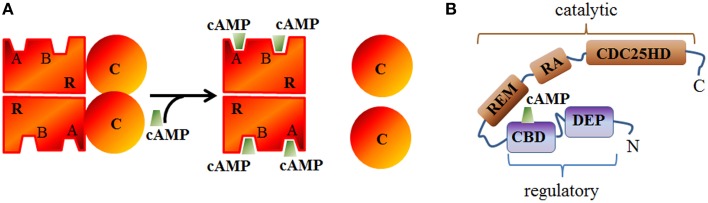
**Schematic structure of PKA and EPAC. (A)** The catalytic (c) subunit of cAMP-dependent Protein Kinase (PKA) is a serine/threonine protein kinase associated, in the absence of cAMP, with the regulatory (R) subunit to form the inactive PKA holoenzyme. cAMP can bind to A or B sites in the regulatory subunits and induces the dissociation of the catalytic subunits. **(B)** Epac structure showing the conserved cAMP binding domain (CBD), Disheveled/Egl-10/pleckstrin (DEP) domain, RAS exchange motif (REM) domain, RAS association (RA) domain, and CDC25-homology domain (CDC25HD).

Inactive PKA relies on an heterotretamer consisting of two regulatory (R) and two catalytic (C) subunits (Figure 3A). Two principal isoforms of the R-subunit (type I and II) each further subclassified into α and β subtypes (Hofmann et al., 1975) and three isoforms of the C-subunit have been described in mammals (Cα, Cβ, and Cγ) (Uhler et al., 1986; Beebe et al., 1990). RIIα is the major isoform expressed in the heart but can also be found in the brain (Skalhegg and Tasken, 2000). RIα is also expressed in the cardiac tissue and the central nervous system while RIβ and RIIβ are respectively found in the spinal cord or brain and liver or fat tissue (reviewed in Skalhegg and Tasken, 2000). In regard to its molecular mechanism of activation, two cAMP molecules bind to each R-subunit and induce a conformational rearrangement of PKA which initiates the functional dissociation of the regulatory from the catalytic subunits (Murray, 2008).

The classically used PKA inhibitors, H89 (isoquinolone derivative) and KT5720 (synthesized from fungus *Nocardiopsis* sp.) act as competitive antagonists of the cAMP nucleotide for the binding site on the PKA regulatory subunit (Kase et al., 1987; Engh et al., 1996). Studying the specificity for commonly used inhibitors for a range of protein kinases, Davies and coworkers found unspecific effect for H89 and KT5720 as they were found to inhibit other kinases at lower concentrations than those used to prevent PKA activation (Davies et al., 2000). For instance, H89 is able to inhibit ROCK, S6K, PKBα, or MSK1 while KT5720 inhibits PDK1 and PHK (Davies et al., 2000). Alternate PKA inhibitors were developed including Rp-cAMPs and its derivatives. Those inhibitors act as competitive antagonists of the cyclic nucleotide binding domain on the regulatory PKA subunit. A study in *Dictyostelium* has characterized both selectivity and degradation of such compounds (Schaap et al., 1993) and demonstrated that those molecules can indeed inhibit proteins containing other cAMP binding domain. Finally, the protein kinase inhibitor peptide (PKI) remains likely the most specific way to interfere with PKA as it binds to the free catalytic subunit and prevents phosphorylation of PKA targets (Dalton and Dewey, 2006). However, high concentration of this peptide can also inhibit PKG signaling (Glass et al., 1992). By opposition to PKA inhibitors, 8pcpt-cAMP and its derivative (Sp-5,6-DCl-cBiMPS) are cell permeable cAMP analogs that can bind the PKA-cAMP binding site and promote the activation of PKA downstream effectors (Sandberg et al., 1991).

#### Epac inhibitors and activators

Epac (exchange protein activated by cAMP) constitutes with PKA the main direct cAMP effector and has been identified by two independent group in 1998 (de Rooij et al., 1998; Kawasaki et al., 1998). In mammals, two isoforms of Epac (Epac1 and Epac2), products of independent genes have been identified which contain a cAMP binding domain (that is homologous to that of PKA R subunits) and other conserved domains (Figure 3B). Its activation relies on a conformational rearrangement of the protein promoted by cAMP binding. Epac1 is mostly abundant in the heart, kidney, blood vessels, adipose tissue, central nervous system, ovary, and uterus, whereas Epac2 splice variants (Epac2A and Epac2B) are mostly expressed in the central nervous system (Epac2A), adrenal gland (Epac2B), and pancreas (Epac2A) (de Rooij et al., 1998; Kawasaki et al., 1998; Niimura et al., 2009). Once activated, Epac proteins activate the Ras superfamily small G proteins Rap1 and Rap2 (for review Cheng et al., 2008) by functioning as guanine nucleotide exchange factors. In cardiomyocytes, Epac proteins are involved in the formation of gap junctions to coordinate cardiac contractions through gating ions and small molecules (Somekawa et al., 2005) and enhances intracellular Ca^2+^ release during cardiac excitation-contraction coupling (Pereira et al., 2007). A High throughput screening assay to identify Epac inhibitors without affecting PKA activity led Cheng and coworkers to the identification of ESI-05 as an isoform specific inhibitor of Epac2 but not Epac1 (Tsalkova et al., 2012) and ESI-09 an pan inhibitor of Epac1 and 2 (Almahariq et al., 2013). CE3F4 compound (Courilleau et al., 2012) was identified as a specific Epac1 inhibitor without influence on PKA activity and its isoform selectivity for EPAC1 toward EPAC2 was demonstrated later (Courilleau et al., 2013). CE3F4 could be of interest in the therapeutic of cardiac pathophysiology as Epac1 is involved in β-adrenergic receptor-induced cardiomyocyte hypertrophy (Métrich et al., 2008). The compound usually named 007 [8-(4-Chloro-phenylthio)-2'-O-methyl-cAMP] is a cAMP analog activating Epac but not PKA (Enserink et al., 2002) but it has to be noted that 007 can behave as an inhibitor of PDEs which may indirectly increases cyclic nucleotide concentration (Poppe et al., 2008).

#### CNGC inhibitors

The family of cyclic nucleotide gated channels (CNGC) comprises two groups: cyclic nucleotide gated (CNG) channels and the hyperpolarization-activated cyclic nucleotide-gated (HCN) channels. Both types are members of the six transmembrane channel superfamily and contain a cyclic nucleotide binding domain in their cytosolic C-Terminus that serves as an activation domain. Upon cyclic nucleotide binding, CNG channels gates the flow of monovalent cations such as Na^+^ and K^+^ to cross the plasma membrane and have a greater sensitivity for cGMP than for cAMP (for review Podda and Grassi, 2014). HCN cations channels open upon hyperpolarization and cAMP enhance their activity by shifting the activation curve to more positive voltage (Scicchitano et al., 2012). Four members exist in mammals (HCN1–HCN4) and are known to regulate the If current to control heart rate and rhythm by acting as a pacemaker current in the sinoatrial node (Wahl-Schott et al., 2014). If current is regulated by various neurotransmitters and metabolic stimuli (Pape, 1996) and are promising pharmacological targets in the treatment of cardiac arrhythmias. Thus, the most extensively studied HCN channels blocker is ZD7288 (BoSmith et al., 1993) but If current can also be blocked by ivabradine (Bucchi et al., 2002, 2006), zatebradine, and cilobradine (Van Bogaert and Pittoors, 2003). Ivabradine derivatives led to the discovery of HCN selective blockers with EC18 identified as a selective blocker for HCN4 and MEL57A induced mHCN1 inhibition (Melchiorre et al., 2010; Del Lungo et al., 2012).

#### POPDC inhibitor

The Popeye domain-containing gene family consists of 3 genes (podc1, podc2, and popdc3) encoding a 3 transmembrane proteins that bind cAMP through their conserved cytoplasmic Popeye domain with an affinity (IC_50_) of 120 nM, which is comparable to the affinities reported for PKA (100 nM) (Froese et al., 2012). These proteins are essential for stress mediated modulation of cardiac pacemaking (Froese et al., 2012; Schindler et al., 2012). To our knowledge, no pharmacological inhibitors have been reported to investigate specific POPDC protein function so that the only way to modulate their activities so far is the use of genetic tools (small interfering RNA technology or gene knockout).

## Methods for cAMP detection in the cardiac tissue

The number of technologies that enables the functional screening of cAMP production has expanded over the years. Consequently, the choice of the technology will define the scope of the conclusions that can be drawn. Those methods can be divided into two groups: the direct methods allowing an “absolute” cAMP concentration quantification and the indirect methods which give a relative representation of cAMP availability. Thus, as summarized in Table 3, direct methods are generally more sensitive than indirect one since lacking any mediator but cannot accurately sense low cAMP levels produced in subcellular compartments. The advantages and limits of the common systems are listed below and basic principle for each technique is shown on Table 3. As previously pinpointed, cAMP availability is fine-tuned by a tight balance between its synthesis and immediate hydrolysis/efflux/use so that at one time point, cAMP is not enough amenable to quantification assays. Thus, accumulation of cAMP is often mandatory in most of cAMP detection assays with the common use of the pan PDEs inhibitor IBMX (when cAMP production needs to be measured) or FSK pretreatment (when cAMP production inhibitory function wants to be outlined).

**Table 3:** **Comparison of cAMP detection system**.

**Class**	**Method**	**Principle**	**Localization**	**Sensitivity**	**Signal in presence of cAMP**
**Biochemical**	Radiometric	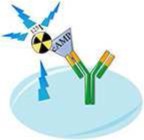	Homogenate	10 nM	↘
	Fluorescence polarization	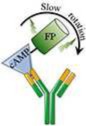	Homogenate	10 nM	↗
	Enzyme or fluorescence detection	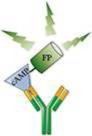	Homogenate	1 nM	↘
	HTRF	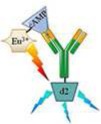	Homogenate	1 nM	↘
	AlphaScreen	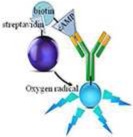	Homogenate	1 nM	↘
	Enzyme complementation	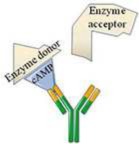	Homogenate	1 nM	↗
	Electroluminescence	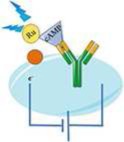	Homogenate	1 nM	↘
**Integrative**	cAMP binding on circularly permutted luciferase	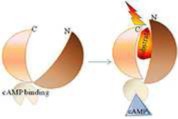	Whole cell	100 μM	↗
	CNGC based	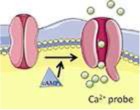	Whole cell	nd	↗
**Reporter gene**	CRHB response element	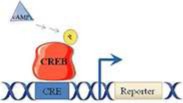	Whole cell	nd	↗
**RET Based**	PKA based	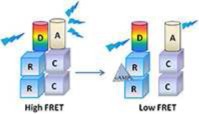	Cytosol		↘
	Epac based	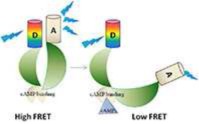	Plasma membrane Endosome Cytosol		↘ ↘ ↘
	CNGC based	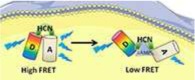	Membrane Membrane Membrane	0.1 μM 1 μM 50 μM	↘ ↘ ↘
**Click chemistry**	Copper free Azide-alkaline cycloaddition		Derivative cAMP syntesis	nd	↗

### Direct methods: Biochemical approaches

cAMP has been long quantified through a radioactive functional assay based on affinity chromatography purification using ^3^H-ATP-preloaded cells lysates. After lysis and cAMP production can be estimated by measuring the ratio between ^3^H-cAMP over ^3^H-ATP + ^3^H-cAMP separated on affinity column (Piñeyro et al., 2005). Despite its high sensitivity, this technique often requires the presence of PDEs inhibitor and is not suitable for high throughput screening (HTS) strategy. Most of HTS biochemical methods relies on the general principle that cAMP accumulation is being detected by competition for a specific cAMP antibody between free unlabelled cAMP present in the sample to evaluate and a labeled form (radioactive, fluorescent, or enzymatic) of cAMP (Williams, 2004). Radiometric assays allow detection of cAMP using competition with ^125-^I labeled cAMP for anti-cAMP antibody immobilized on a solid scintillant plate. In those assays, the radiometric signal decreases proportionally to the amount of cAMP present in the sample (Horton and Baxendale, 1995). Fluorescence polarization cAMP assays monitor the light emitted from a fluorescent-tagged cAMP following excitation by a polarized light source. When the labeled cAMP is bound to an antibody more polarized light will be produced upon excitation (Prystay et al., 2001; Huang et al., 2002). To increase the signal to noise and to avoid cell autofluorescence detection, Cisbio developed an HTRF (Homogeneous time resolved fluorescence)-based cAMP assay. This assay is still based on a competitive immunoassay using cryptate-labeled anti-cAMP antibody and d2-labeled cAMP (Degorce et al., 2009). Alpha-screen technology uses acceptor beads conjugated to an antibody that recognizes cAMP and streptavidin-coated donor beads. When brought into close proximity by the presence of biotinylated cAMP, an oxygen radical dependent light is emitted. The cAMP extracted from a cell lysate will compete with the biotinylated cAMP and reduce the emitted light. This kind of assay is also available with an enzymatic based detection method where cAMP found in test sample competes with a fixed amount of Horse Radish Peroxydase-linked cAMP for binding to an anti-cAMP immobilized antibody (Bouchard et al., 2006). The electroluminescence technique (Mesa Scale Discovery) is another competitive immunoassay based on the displacement of ruthenium-labeled cAMP for an anti-cAMP antibody. The electrochemical reaction is initiated upon substrate and electrical charge addition and produce light which is inversely proportional to the cAMP present in the sample (Filip et al., 2004). The immuno-based competition can also be revealed using an enzyme complementation method (DiscoverX). In this assay, a fragment of β-galactosidase (β-gal) is conjugated with cAMP and act as an enzyme donor (ED) (Golla and Seethala, 2002). This ED-cAMP conjugate and cellular cAMP compete for binding to an anti-cAMP antibody. In presence of the enzyme acceptor (EA), the active enzyme will be reconstituted and will be able to subsequently hydrolyze a substrate to produce a chemiluminescent signal that is directly proportional to the amount of cAMP in the cells. Despite being sensitive and specific, all those techniques require cells or tissue disruption making the real-time and sub-cellular analysis of cAMP quantification impossible. Moreover if those assays are highly efficient to measure cAMP production, their use to bring to the fore cAMP inhibition is challenging and require FSK pretreatment.

### Indirect methods

#### Integrative methods

Promega developed an assay based on the GloSensor Technology, a genetically modified form of firefly luciferase into which a cAMP-binding protein domain has been inserted (Fan et al., 2008). The firefly luciferase is circularly permuted and upon cAMP binding, a conformational change induces luciferase enzyme reconstitution which produces light in presence of its substrate. This technique is sensitive enough to assess role of endogenous receptors but requires transfection of the biosensor, thus limiting the quantification of the cAMP to the transfection efficiency (expression heterogeneity between cells) which can lead to high results variability. Maintaining advantages of integrative methods (sensitivity and kinetic) but avoiding transfection variability, Rivero-Müller's group developed a derived detection method CANDLES (Cyclic AMP iNdirect Detection by Light Emission from Sensor cells). Briefly, a stable cell line expressing a GloSensor plasmid is co-cultured with cells expressing the receptor to be tested and through cell-cell interaction via gap junctions, cAMP produced by the cell of interest can be transferred to the sensor cells to activate GloSensor plasmid (Trehan et al., 2014). Finally, given that the GloSensor is a cytosolic probe, it could be not appropriate to detect low concentrations of compartmentalized cAMP at the plasma membrane.

#### Reporter gene methods

The reporter gene method is a homogeneous, simple and inexpensive but indirect method to detect cAMP-downstream signaling. This assay is based on the specific activation of the transcription factor CREB (cAMP response element binding protein) upon cAMP production which induces a reporter gene under the control of a CRE element (cAMP Response Element) promoter. Various reporter genes have been used over the years: fluorescent proteins, luciferase, β-galactosidase or β-lactamase. Far downstream of the cAMP activation cascade, this method is sensitive but unable to give kinetics or localization information.

#### Resonance energy transfer methods

The methods described above are unable to define the cellular localizations of cAMP production. Thus, visualization was achieved using resonance energy transfer (RET) techniques described by the Theodor Förster in 1940's (Forster, 1946). RET is a mechanism relying on an energy transfer between a donor chromophore that may transfer energy to another acceptor chromophore through non-radiative dipole–dipole coupling upon distances proximity conditions (Hebert et al., 2006; Kiyokawa et al., 2006). The name FRET “Förster resonance energy transfer” which includes the commonly used term FRET “Fluorescence resonance energy transfer” and BRET “Bioluminescence resonance energy transfer” is a non-radiative transfer of energy occurring between two fluorescent chromophores for FRET or between an enzyme generating luminescent signal upon addition of its substrate and a fluorescent acceptor partner in the case of BRET technology. The efficiency of this energy transfer is inversely proportional to the sixth power of the distance between donor and acceptor, making RET extremely sensitive to very small changes in distance thus allowing an accurate sensing of change in protein conformations for instance (Hebert et al., 2006). BRET is a first line assay for HTS screening as it avoids the consequences of fluorescence excitation and has a better Stokes' shift over FRET but is not recommended for imaging technique to identify localized cAMP compartmentalization. Thus RET-based methods have been developed to detect cAMP production and extensively reviewed (Williams, 2004; Willoughby and Cooper, 2008; Sprenger and Nikolaev, 2013; Calebiro and Maiellaro, 2014). All the methods rely on the use of the downstream cAMP effectors PKA, Epac or CNGC either that all directly bind cAMP molecules related to the expression of a specific cAMP-binding motif. Briefly, either full-length cAMP-effector probes or single cAMP domain sensor extracted from the different effectors are fused to an energy donor and acceptor allowing the generation of a basal RET signal in the absence of cAMP production. Upon cAMP binding, a conformational rearrangement in the RET-based sensor will lead to a modification of the RET signal. Since the pioneering studies using those RET probes to study cAMP availability in cardiac tissue (Zaccolo et al., 2000; Zaccolo and Pozzan, 2002), many efforts have been made these last years to improve signal to noise ratio, RET efficiency (optimizing donor-acceptor couple, linker optimization), cAMP binding affinity (mutagenesis on single domain or full length protein probes), RET detection methods (e.g., Sensitized emission vs Fluorescence lifetime imaging microscopy for FRET-based probes, Renilla luciferase variants for BRET-based probes) (Willoughby and Cooper, 2008; Sprenger and Nikolaev, 2013). Moreover with the prominently recognized mechanism for cAMP compartmentation, several group restricted the expression of those RET based probes to subcellular localization using for example plasma membrane targeting sequence or endosome localization (Klarenbeek and Jalink, 2014; Sprenger et al., 2015).

#### Copper free azide-alkaline cycloaddition: a “Click Chemistry”

In chemical synthesis, click chemistry is a process that generates by joining small units together. The azide alkyne Huisgen cycloaddition using a Copper (Cu) catalyst is one of the most popular reactions within the Click chemistry concept between an azide and a terminal or internal alkyne to give a 1,2,3-triazole (Rostovtsev et al., 2002; Tornøe et al., 2002). To avoid Cu toxicity, Baskin *et al* developed a Cu-free click reaction with comparable kinetics to Cu dependent cycloaddition, but adapted for dynamic *in vivo* imaging (Baskin et al., 2007). In a recent study, this copper free method was applied to detect cAMP derivative (8-azido cAMP) *in vivo* after the addition of difluorinated cyclooctyne (DIFO) as a reagent (Ito et al., 2013). If this approach will enable to visualize and quantify derivative cAMP endogenous modulators at the single cell level without exogenous transfection protocol, it has to be noted that the molecule used is a cAMP derivative so the signal observed will be the result of ACs/PDEs activities in competition with cAMP endogenously produced.

## Conclusion

**Figure 4:**
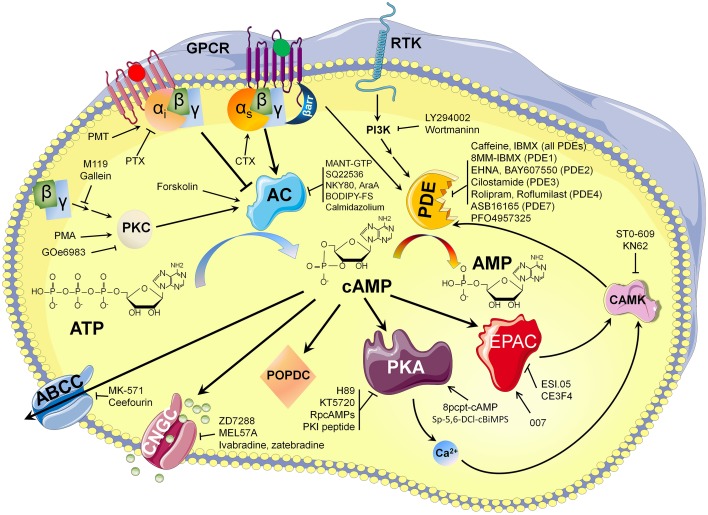
**cAMP synthesis and hydrolysis: pharmacological way to modulate its availability**.

The development of optical methods that allow monitoring of cAMP dependent signaling in living cells and the growing list of molecules (summarized in Figure 4) available to modulate cAMP availability played a fundamental role in revealing an unexpected level of cAMP organization in cardiac tissue. It is likely that new optical methods development, with higher temporal and spatial resolution, will improve our knowledge of cAMP dependent signaling microdomains located on the cell surface or other intracellular membranes for individual cells within heart architecture.

## Funding

This work was supported and funded by “Fondation Bettencourt Schueller and Institut National de la santé et de la Recherche Médicale.”

### Conflict of interest statement

The authors declare that the research was conducted in the absence of any commercial or financial relationships that could be construed as a potential conflict of interest.

## Footnotes

^1^http://www.informatics.jax.org/marker/MGI:2660854. Informatix JAX.

^2^http://www.informatics.jax.org/marker/MGI:97523. JAX.

^3^http://www.informatics.jax.org/reference/marker/MGI:108413. JAX.
